# Preliminary Assessment of Wind and Wave Retrieval from Chinese Gaofen-3 SAR Imagery

**DOI:** 10.3390/s17081705

**Published:** 2017-07-25

**Authors:** Weizeng Shao, Yexin Sheng, Jian Sun

**Affiliations:** 1 Marine Science and Technology College, Zhejiang Ocean University, Zhoushan 316000, China; shaoweizeng@zjou.edu.cn (W.S.); shengyexin@hotmail.com (Y.S.); 2 Key Laboratory for Earth Observation of Hainan Province, Hainan 572029, China; 3 Physical Oceanography Laboratory/CIMST, Ocean University of China and Qingdao National Laboratory for Marine Science and Technology, Qingdao 266100, China

**Keywords:** Gaofen-3, synthetic aperture radar, wind, wave

## Abstract

The Chinese Gaofen-3 (GF-3) synthetic aperture radar (SAR) launched by the China Academy of Space Technology (CAST) has operated at C-band since September 2016. To date, we have collected 16/42 images in vertical-vertical (VV)/horizontal-horizontal (HH) polarization, covering the National Data Buoy Center (NDBC) buoy measurements of the National Oceanic and Atmospheric Administration (NOAA) around U.S. western coastal waters. Wind speeds from NDBC in situ buoys are up to 15 m/s and buoy-measured significant wave height (SWH) has ranged from 0.5 m to 3 m. In this study, winds were retrieved using the geophysical model function (GMF) together with the polarization ratio (PR) model and waves were retrieved using a new empirical algorithm based on SAR cutoff wavelength in satellite flight direction, herein called CSAR_WAVE. Validation against buoy measurements shows a 1.4/1.9 m/s root mean square error (RMSE) of wind speed and a 24/23% scatter index (SI) of SWH for VV/HH polarization. In addition, wind and wave retrieval results from 166 GF-3 images were compared with the European Centre for Medium-Range Weather Forecasts (ECMWF) re-analysis winds, as well as the SWH from the WaveWatch-III model, respectively. Comparisons show a 2.0 m/s RMSE for wind speed with a 36% SI of SWH for VV-polarization and a 2.2 m/s RMSE for wind speed with a 37% SI of SWH for HH-polarization. Our work gives a preliminary assessment of the wind and wave retrieval results from GF-3 SAR images for the first time and will provide guidance for marine applications of GF-3 SAR.

## 1. Introduction

The Chinese Gaofen-3 (GF-3) satellite was launched on 10 August 2016 by the China Academy of Space Technology (CAST), and carries a C-band (~5.3 GHz) synthetic aperture radar (SAR) sensor with different polarizations. It has 12 imaging modes with a spatial resolution of image ranging from 1 m to 500 m and a swath coverage ranging from 10 to 650 km. GF-3 SAR operates in different polarizations, including single-, dual- and quad-polarization. Through a cooperation project between the National Satellite Ocean Application Service (NSOAS) and our institutes, a number of images have been recorded of whole open seas, particularly covering the National Data Buoy Center (NDBC) in situ buoys of the National Oceanic and Atmospheric Administration (NOAA) around U.S. western coastal areas. Wind and wave monitoring are the two main marine applications of SAR in all-weather conditions, especially in tropical cyclones [1,2].

It is well known that Bragg waves, which have the wavelength with the order of centimeters, backscatter the microwaves of SAR. Bragg waves are produced by sea surface winds. Through studying co-polarization (vertical-vertical (VV) and horizontal-horizontal (HH)) Spaceborne Imaging Radar (SIR) data on Seasat launched in 1978 [3,4], the geophysical model function (GMF) has been exploited. GMF describes an empirical relationship between normalized radar cross-section (NRCS) in VV-polarization and a wind vector. C-band GMFs, e.g., CMOD4 [5], CMOD-IFR developed at Institut Francais de Recherche pour Exploitation de la MER (IFREMER) [6], CMOD5 [7] and CMOD5N [8]. These GMFs provide a convenient application for wind retrieval from SAR [9,10,11] and have been successfully implemented for various C-band SAR data over the last few decades, e.g., ERS-1/2 [12], Envisat-ASAR [13], Radarsat-1/2 [14] and Sentinel-1A/1B [15] within about 2 m/s error of wind speed. CMOD4 and CMOD-IFR work at wind speeds smaller than 20 m/s, due to no higher wind source being available in the tuning process. The formulation of an improved C-band GMF CMOD5 was essentially redesigned with a number of ERS-2 images taken in tropical cyclones and corresponding European Centre for Medium-Range Weather Forecasts (ECMWF) re-analysis winds. In particular, some case studies have shown that CMOD5 can be used in hurricanes [16,17,18] to some extent. Later, CMOD5 was retuned for neutral winds, denoted as CMOD5N, which takes a correction for CMOD5 can be more stable applied. As GMF is applied for HH-polarization SAR, NRCS in HH-polarization has to be converted into NRCS in VV-polarization by using a polarization ratio (PR) model [19,20,21,22,23]. The latest achievement of the PR model was proposed by the authors of [23] and the improvement is that the dependence of sea surface wind speed on PR is included in the model. More recently, using collocated scatterometer measurements on aboard Metop-A/B and ECMWF winds with co-polarization Envisat-ASAR and Sentinel-1A/1B SAR data, a new C-band GMF for wind retrieval from co-polarization SAR was developed in [24], denoted as C-SARMOD. Although the accuracy of wind speeds retrieved from Sentinel-1A/1B images in VV-polarization by using C-SARMOD showed a 1.6 m/s STD of wind speed against moored buoy measurements [25], the validation of its application for C-band SAR in HH-polarization has not yet been systematically investigated.

Traditionally, the methodology of wave retrieval from SAR is based on the SAR mapping mechanism [26]. The major modulations of waves on SAR have been well studied over many decades, e.g., tilt modulation, hydrodynamic modulation and velocity bunching [27,28]. Both tilt and hydrodynamic modulations are linear mapping mechanisms and the two modulation transfer functions (MTF) were proposed by the authors of [29,30]. However, velocity bunching is caused by the relative motion between satellite platform and sea surface, resulting in a Doppler frequency shift in the azimuth direction (the direction of satellite flight is defined as the azimuth direction and radar look direction is the range direction). This velocity bunching causes wavelengths smaller than a specific value (or cutoff wavelength) in the azimuth direction, which is not detectable and the peak of the SAR spectrum rotates toward the range direction. These two effects create the difficulty of directly inverting SAR intensity spectra to wave spectra. The first solution was established by the authors of [28], with an algorithm known as the Max–Planck Institute (MPI) algorithm. The basic scheme of the MPI is described as follows: (1) a first-guess wave spectrum is produced from numeric wave modes, e.g., Simulating WAves Nearshore (SWAN) and WaveWatch-III; (2) a simulated SAR spectrum is obtained by mapping the first-guess wave spectrum; (3) the reality of the wave spectrum is inverted by minimizing the simulated and real SAR spectrum through a cost function. The Semi Parametric Retrieval Algorithm (SPRA) [31] is more applicable than the MPI, because the SPRA employs wind measurements from a scatterometer in order to produce the first-guess spectrum by using the Jonswap parametric model. Later the Parameterized First-guess Spectrum Method (PFSM) algorithm was proposed by the authors of [32,33] and recent studies show that the PFSM works for X-band TerraSAR-X [34] and C-band Sentinel-1 SAR data [25]. The improvement of the PFSM involves the wind-wave and swell information on SAR being separated by calculating the wave number threshold of the SAR intensity spectra. A 0.54 m standard deviation (STD) of SWH is exhibited in study [25] by using the algorithm PFSM as the SAR-derived SWH from Sentinel-1 SAR data validated against the buoys. In addition, there are other wave algorithms, e.g., the Partition Rescaling and Shift Algorithm (PARSA) [35] for SAR complex data and unconstrained algorithms [36,37], in which the MTF of velocity bunching is bypassed and it only works for long wave dominated regions.

Empirical wave retrieval algorithms, without calculating complex MTF, were developed by the SAR group at the German Aerospace Center (DLR), and include CWAVE_ERS for ERS-2 SAR [38], CWAVE_ENV for Envisat-ASAR [39] and XWAVE for X-band TerraSAR-X SAR [40]. CWAVE allows direct retrieval of significant wave height (SWH) from SAR wave mode data at a fixed incidence angle of about 23°. However, these are not conducive to the operational application of wave retrieval from various SAR data. Interestingly, the cutoff wavelength in azimuth direction caused by velocity bunching is theoretically related with SWH [28]. Several recent studies have made great efforts to retrieve wave parameters through the cutoff wavelength [41,42,43,44,45]. In our previous study [46], the four existing algorithms, including PFSM and three other empirical algorithms, have been implemented for HH-polarization Sentinel-1 SAR images. It was found that the empirical algorithm, herein called CSAR_WAVE, has a good performance as the retrieval results compared with moored buoys.

In this study, we give a preliminary assessment of wind and wave retrieval from the new Chinese C-band GF-3 SAR for the first time. After employing ECMWF wind direction, wind speed is retrieved from a VV-polarization GF-3 SAR image using CMOD5N. The PR model proposed in [23] is used together with CMOD5N for wind speed retrieval from an HH-polarization GF-3 SAR image. SWH is retrieved using CSAR_WAVE for both VV and HH polarization GF-3 SAR without any prior knowledge.

The remainder of this paper is organized as follows. The description of collected C-band co-polarization GF-3 SAR images and the validation sources, including NOAA buoy measurements, available ECMWF re-analysis winds and wave computations from the WaveWatch-III model provided by the IFREMER group, are briefly introduced in Section 2. Section 3 shows the methodology of wind and wave retrieval algorithms used in this study. Then retrieval results and discussions are presented in Section 4 and Section 5, respectively. Conclusions are summarized in Section 6.

## 2. Description of Datasets

**Figure 1:**
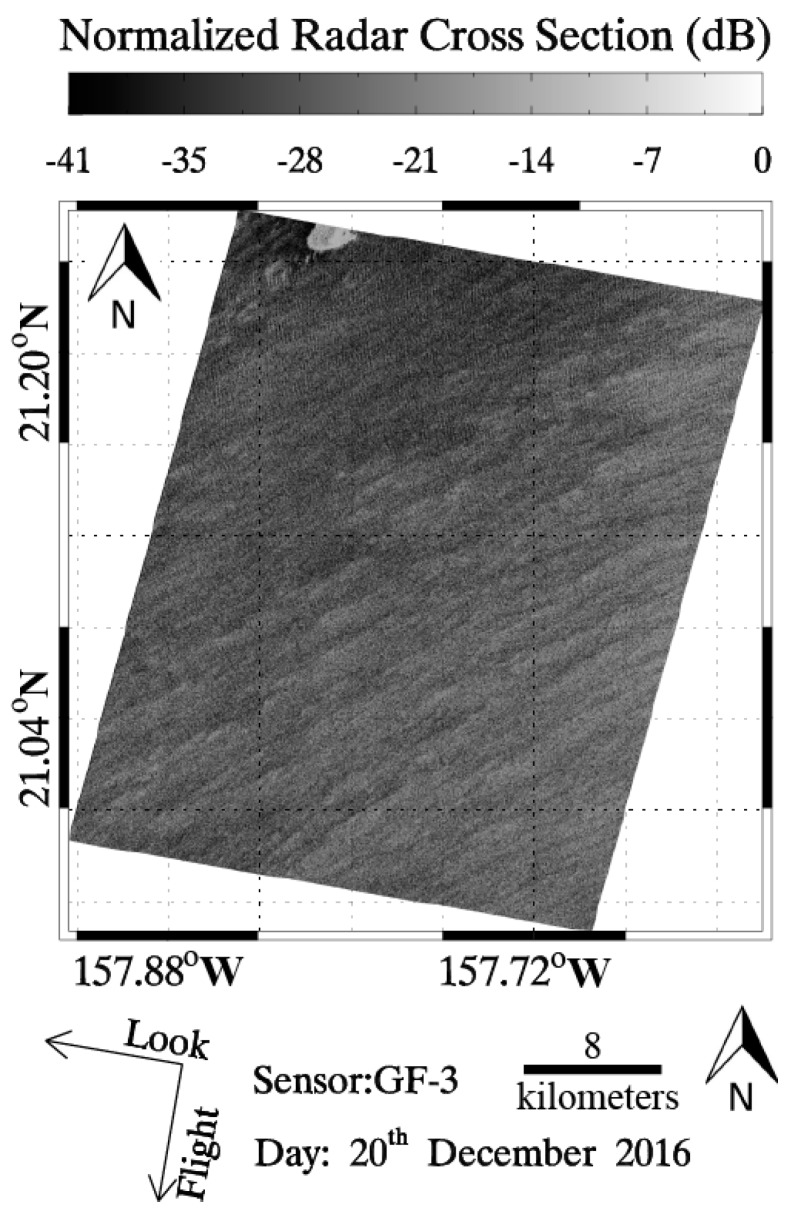
The quick-look image of calibrated Gaofen-3 (GF-)3 image in vertical–vertical (VV)-polarization around the Hawaiian islands acquired in Quad-Polarization Stripmap (QPS) mode at 16:22 UTC on 20 December 2016.

**Figure 2:**
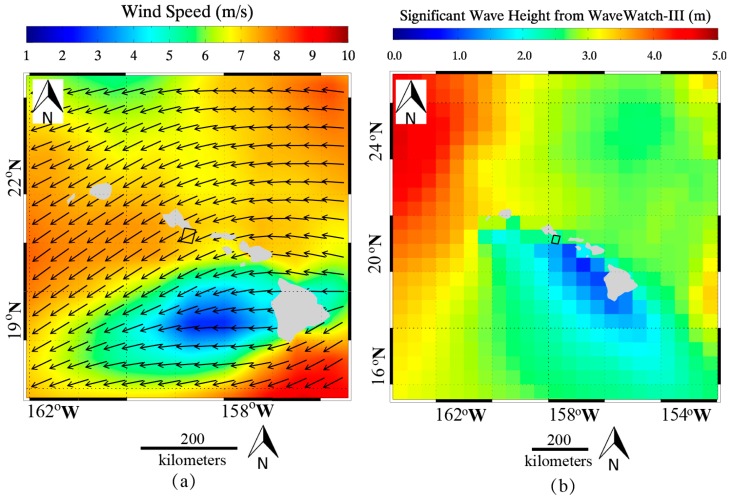
(**a**) European Centre for Medium-Range Weather Forecasts (ECMWF) wind field at 18:00 UTC and; (**b**) Significant wave height (SWH) field from WaveWatch-III at 18:00 UTC on 20 December 2016. The black rectangle represents the coverage of GF-3 synthetic aperture radar (SAR) image in Figure 1.

In total, we collected 224 GF-3 SAR images in co-polarization (VV and HH polarization) through September 2016 to March 2017 at seas. These single look complex (SLC) images were acquired in Stander Stripmap (SS) or Quad-Polarization Stripmap (QPS) mode. In the 224 images, there are 166 images, which are a matchup with 0.125 × 0.125° grids ECMWF re-analysis winds at intervals of six hours and 0.5 × 0.5° grids waves from the WaveWatch-III model at intervals of 3 h. The time difference between the imaging time of those images and ECMWF/WaveWatch-III data was within two hours. They were used to investigate the accuracy of wind and wave retrieval results in our study. The following equation is used for calculating the NRCS of co-polarization GF-3 SAR intensity image.

(1) $$\sigma^{0}={\mathrm{DN}}^{2}{\left(\frac{M}{32767}\right)}^{2}-N$$

wherein σ° is the NRCS united in dB, DN is the intensity derived from GF-3 SAR Level-1A data, M is the external calibration factor and N is the offset constant stored in the annotation file. As an example, a quick-look image of a calibrated GF-3 SAR image in VV-polarization around the Hawaiian islands acquired in QPS mode at 16:22 UTC on 20 December 2016 is shown in Figure 1. The ECMWF wind field at 18:00 UTC is shown in Figure 2a and SWH from the WaveWatch-III model at 18:00 UTC are shown in Figure 2b. The black rectangle represents the coverage of the case image. In the matchups, we only used homogenous sub-scenes derived from images for wave validation, that is, the good-quality SAR intensity spectra can be obtained by using the two-dimensional Fast Fourier Transform (FFT) method.

Out of the 166 GF-3 SAR image matchups, 58 images cover the NDBC in situ buoys of NOAA around U.S. western coastal areas. It is necessary to calculate that the wind speeds measured by the NDBC in situ buoys are at a height of 5 m above the sea surface, so we use Equation (2) to convert buoy-measured wind speeds to values at 10 m height as neutral winds,

(2) $$\frac{U_{10}}{U_{5}}\;=\;\frac{\ln(10/z_{0})}{\ln(5/z_{0})}$$

wherein U_10_ is the wind speed at 10 m height, U_5_ is the wind speed measured by NDBC in situ buoy and z_0_ is the roughness length taken as a constant 1.52 × 10^−4^ [25,46].

## 3. Wind and Wave Retrieval Algorithms for C-Band SAR

In this section, the methodology of wind and wave retrieval from SAR is presented, including the C-band GMF with PR model for wind retrieval and a cutoff-wavelength-based empirical algorithm for wave retrieval.

### 3.1. Wind Retreival Algorithm

Until now, the CMOD family has successsfully been applied for wind retreival from various C-band SAR data [9,10,11]. The CMOD family takes the general formulation:

(3) $$\sigma^{0}=B_{0}{\left(1\;+\;B_{1}\cos\phi \;+\;B_{2}\cos2\phi \right)}^{p}$$

wherein σ° is the linear SAR-measured NRCS, ϕ is the wind direction related to range direction, p is a parametric constant, B_0_ to B_2_ are the functions of wind speed at 10 m above sea surface U_10_ and radar incidence angle is θ. CMOD5N is the latest version and the accuracy of wind speeds retrieved from a number of Sentinel-1 SAR images using CMOD5N have been investigated in [15], showing a 1.35 m/s STD of wind speed validated against scatterometer measurements on aboard Metop-A/B. Because there are two unknown variables, e.g., wind speed and wind direction, it is impossible to solve Equation (3) to invert the wind vector. It has been found that homogenous wind streaks at the kilometer scale, which are parallel to the wind direction, can be retrieved from some SAR images [47]. However, a SAR-derived wind direction has a 180° ambiguity and external information is required so as to remove that ambiguity. Moreover, wind streaks do not appear in SAR images. Therefore, we directly employed ECMWF wind directions in the wind retrieval process.

PR models, which are described as the ratio between NRCS in VV and HH polarization, are usually used together with CMODs as wind retrieval for C-band HH-polarization SAR [19,20,21,22,23]. Recent research has shown that C-band SAR PR has a linear relation with wind speed [23], in addition to the incidence angle. It is not surprising that the PR model, which includes dependence of sea surface wind speed, performs better than other PR models involving only the dependence of incidence angle, which is stated as follows:

(4) $$\mathrm{PR}\;=\;\frac{\sigma_{\mathrm{VV}}^{0}}{\sigma_{\mathrm{HH}}^{0}}{\;=\;P(\theta )U}_{10}^{Q(\theta )}$$

where

(5) $${P(\theta )\;=\;P}_{1}\theta^{2}{\;+\;P}_{2}{\theta \;+\;P}_{3}$$

and

(6) $${Q(\theta )\;=\;Q}_{1}{\theta \;+\;Q}_{2}$$

wherein $\sigma_{\mathrm{VV}}^{0}$ and $\sigma_{\mathrm{HH}}^{0}$ are the SAR-measured linear NRCS in VV- and HH-polarization respectively, coefficients P_1_ to P_3_ and Q_1_ to Q_2_ are tuned by an amount of quad-polarization Radarsat-2 images and the collocated buoy measurements. The dependence of wind speed on X-band PR has been investigated through dual-polarization TerraSAR-X images and ECMWF re-analysis winds [48] following the PR model proposed by the authors of [23], which was adopted for X-band SAR.

### 3.2. Wave Retreival Algorithm

As introduced in Section 1, the many SAR mapping mechanism-based algorithms [28,30,31,32,33,34,35,36] need a prior ‘first-guess’ wave spectrum provided from the numeric wave mode or produced by the parametric wave function using wind speed. Therefore, none of them can be operationally applied for wave retrieval from SAR. Although the two empirical algorithms, e.g., CWAVE_ERS [39] and CWAVE_ENV [40], work well for SAR data at a specific incidence angle of about 23°, they have yet to be validated for SAR data in imaging mode at various incidence angles. Based on the theoretical relationship between cutoff wavelength in azimuth direction and SWH [28,30], a new empirical algorithm, denoted as CSAR_WAVE, was exploited through 93 C-band Stripmap mode Sentinel-1 SAR images in VV-polarization and collocated wave measurements from NDBC in situ buoys [45]. The validation against buoy measurements showed a 18.6% SI of SWH in our previous study. Recently, CSAR_WAVE has been adopted for HH-polarization Sentinel-1 SAR in [46]. Moreover, CSAR_WAVE provides a convenient empirical method to retrieve SWH for C-band SAR, including, but not limited to, StripMap mode data. The functions of CSAR_WAVE are designed as follows,

(7) $$H_{s}\;=\;\left(\frac{\lambda_{c}}{\beta }\right)\left(A_{1}{\;+\;A}_{2}{\sin\theta \;+\;A}_{3}\cos2\varphi \right){\;+\;A}_{4}$$

(8) $$\beta \;=\;\frac{R}{V}$$

wherein H_s_ is the SWH, λ_c_ is the cutoff wavelength in azimuth direction, β is the satellite range-to-velocity parameter, R is the slant range, V is the satellite flight velocity, θ is the radar incidence angle, φ is the wave propagation angle relative to range direction ranging from 0 to 90° and the coefficients A_1_ to A_4_ are determined from the C-band Sentinel-1 SAR image and collocated NDBC in situ buoys [45] and ECMWF re-analysis wave data at 0.125° [46]. It is necessary to figure out that CSAR_WAVE can operate without any prior knowledge. The advantage of CSAR_WAVE is its application can be implemented without using SAR-derived wind speeds. Therefore, wind and wave are simultaneously measured from co-polarization GF-3 SAR data in our work.

## 4. Method and Results

In this study, wind and wave retrieval results from 58 GF-3 images in co-polarization were validated against NDBC in situ buoy measurements around the U.S. western coastal area, including wind speed and SWH. In order to perform the matchup, each GF-3 imagery was divided into several sub-scenes with a spatial coverage of about 3 × 3 km in azimuth and range direction, respectively. We chose the sub-scenes, covering the moored buoys for studying the accuracy of winds and SWH retrieval results from co-polarization GF-3 images.

### 4.1. Validation of Wind Retreival Results

**Figure 3:**
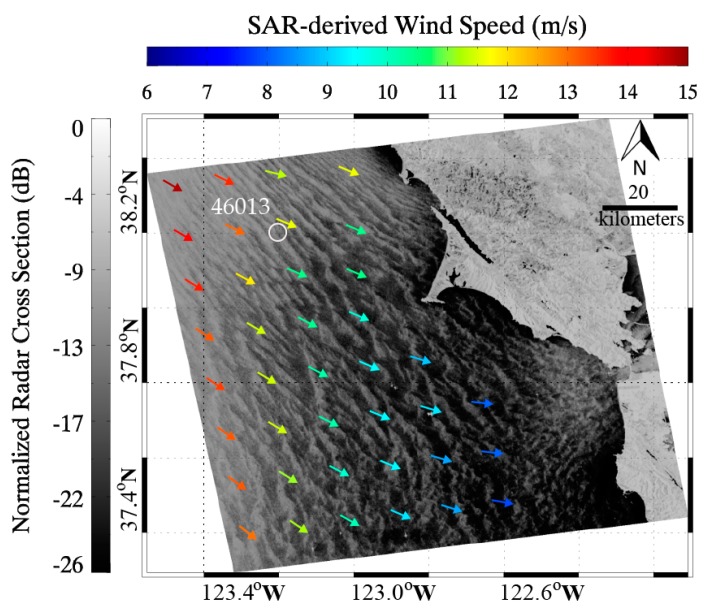
SAR-derived wind field from VV-polarization GF-3 SAR image in Stander Stripmap (SS) mode at 02:17 UTC on 29 September 2016, in which the white circle represents the location of National Data Buoy Center (NDBC) in situ buoy (ID: 46013).

After employing ECMWF wind directions, wind speeds were retrieved from VV-polarization GF-3 SAR images by using CMOD5N. The PR model involving Equations (4)–(6) is used together with CMOD5N for wind speeds retrieval from HH-polarization GF-3 SAR images. As an example, the information retrieved from VV-polarization GF-3 SAR image in SS mode at 02:17 UTC on 29 September 2016 is shown in Figure 3. The SAR averaged wind speed located at the sub-scene of about 3 × 3 km coverage covering the NDBC in situ buoy (ID: 46013) is 11.6 m/s and the buoy-measured wind speed is 10.8 m/s. The difference between the SAR-derived wind speed and the buoy measurement is only 0.8 m/s.

**Figure 4:**
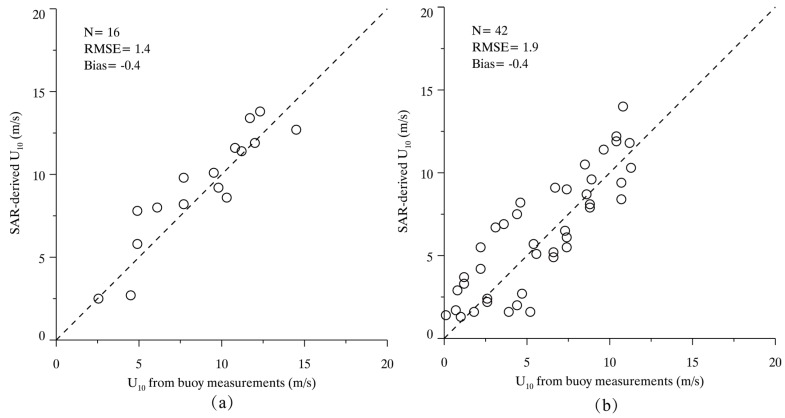
The comparison between SAR-derived wind speed U_10_ and measurements from NDBC buoys. (**a**) Comparison for 16 VV-polarization GF-3 SAR images; and (**b**) Comparison for 42 horizontal–horizontal (HH)-polarization GF-3 SAR images.

We collected a number of sub-scenes from 16/42 GF-3 images in VV/HH polarization matchup with NDBC in situ buoys. The comparison of the matchups is shown in Figure 4. Validation shows a 1.4 m/s/1.9 m/s root mean square error (RMSE) of wind speed with a bias of −0.4 m/s for VV/HH polarization. ECMWF re-analysis winds are popularly used for tuning and validating the wind retrieval algorithms for SAR [7,8]. The accuracy of retrieved wind speeds from co-polarization GF-3 images has a good performance with around 2 m/s RMSE of wind speed. Therefore, it is found that winds retrieval from GF-3 SAR have a similar accuracy to that of the other C-band SARs, which have a up to 1.78 m/s stander deviation (STD) for wind speeds as validated against buoys, scatterometer and numeric models [10,13,14].

### 4.2. Validation of Wave Retreival Results

In this study, we used CSAR_WAVE to retrieve SWH from co-polarization GF-3 SAR images. Two variables, including wave propagation angle relative to range direction φ and cutoff wavelength in azimuth direction λ_c_, are derived from the SAR spectrum. The two-dimensional SAR spectrum is calculated from a SAR intensity image by using the two-dimensional FFT method. φ is directly obtained from a two-dimensional SAR spectrum, which ranges from 0 to 90° in the CSAR_WAVE model. Then, we employ a Gaussian fit function to integrate the two-dimensional SAR spectrum in the range direction. The Gaussian fit function is stated as,

(9) $$\exp\left\{\pi k\times \frac{\lambda_{c}}{2\pi }\right\}$$

in which, k_x_ is the wavenumber in the azimuth direction.

**Figure 5:**
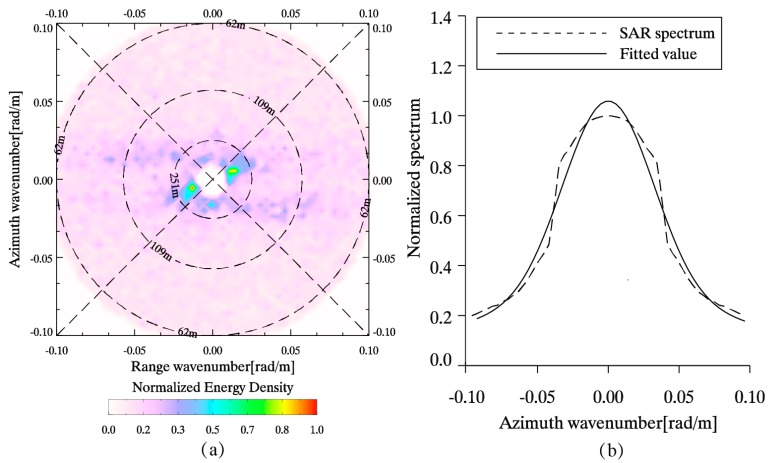
(**a**) The two-dimensional SAR spectrum of sub-scene covering the buoy (ID:46013) in Figure 3; (**b**) The corresponding one-dimensional spectrum with the fitted result by using Gaussian fit function.

**Figure 6:**
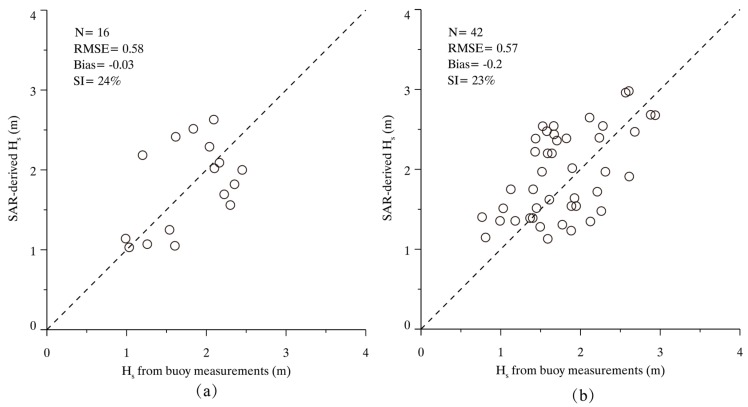
The comparison between SAR-derived H_s_ and the measurements from NDBC buoys. (**a**) Comparison for 16 VV-polarization GF-3 SAR images; and (**b**) Comparison for 42 HH-polarization GF-3 SAR images.

The two-dimensional SAR spectrum of sub-scene covering the NDBC in situ buoy (ID: 46013) in Figure 3 and the corresponding one-dimensional spectrum with the fitted result are shown in Figure 5a,b, respectively. The retrieved SWH is 2.2 m and the buoy-measured SWH is 2.1 m. Figure 6a,b show the further comparisons between SAR-derived SWHs and measurements from NDBC in situ buoys, showing a 24%/23% scatter index (SI) of SWH with a 0.58 m/0.57 m RMSE of SWH for VV/HH polarization.

The retrieval SWH from co-polarization GF-3 SAR using CSAR_WAVE has a close accuracy to that from other C-band SAR by using theoretical-based algorithms and empirical CWAVE models, e.g., about 20% SI of SWH validated against moored buoys or measurements from altimeter [25,38,39] and around 38% SI of SWH as compared to the SWH retrieval results with simulation results from the WAM model [49].

## 5. Discussion

In order to further investigate the accuracy of winds and waves retrieved from GF-3 SAR images. We also compared SAR-derived wind speeds with 0.125 × 0.125° grids ECMWF re-analysis wind speeds and SAR-derived SWH with those from the 0.5 × 0.5° grids WaveWatch-III model through more GF-3 co-polarization images at seas.

**Figure 7:**
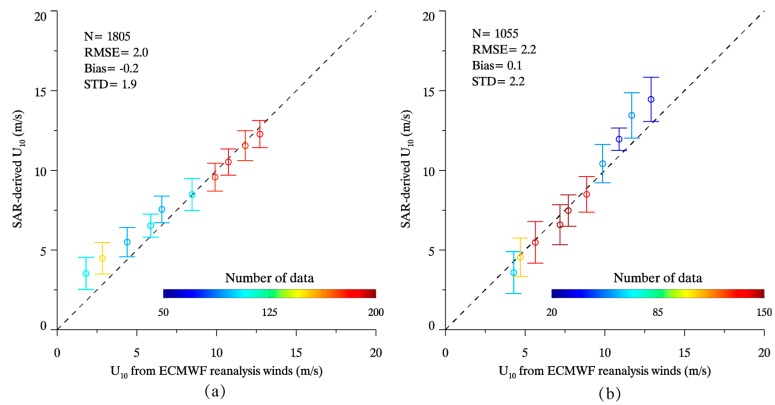
The comparison between SAR-derived wind speed U_10_ and ECMWF re-analysis gridded winds at 1 m/s bins. (**a**) Comparison for 96 VV-polarization GF-3 SAR images; and (**b**) Comparison for 70 HH-polarization GF-3 SAR images.

Out of 58 GF-3 SAR images matchup with buoys, there are additional 166 images in our collections, including 96 images in VV-polarization and 70 images in HH-polarization. The retrieval wind speeds from these images were compared with ECMWF re-analysis gridded winds at 1 m/s bins, showing a 2.0 and 2.2 m/s RMSE of wind speed for VV-polarization and HH-polarization respectively, as exhibited in Figure 7.

**Figure 8:**
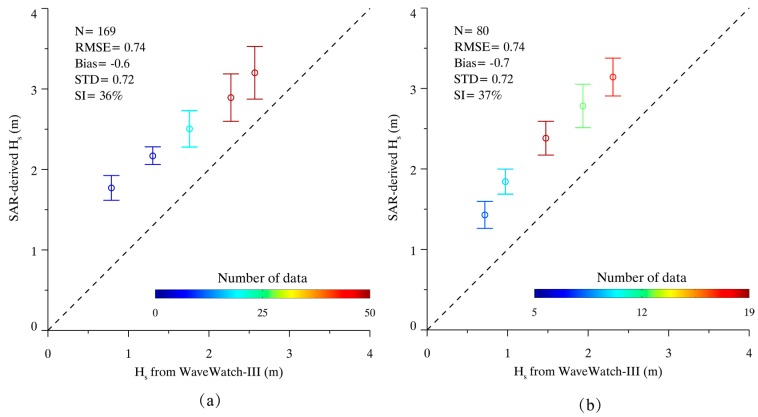
The comparison between SAR-derived SWH H_s_ and WaveWatch-III data at 0.5 m/s bins. (**a**) Comparison of 96 VV-polarization GF-3 SAR images; and (**b**) Comparison of 70 HH-polarization GF-3 SAR images.

The computations run by the WaveWatch-III model at intervals of three hours. In our previous study, that data has been used for the validation of wave retrieval results from X-band SAR by using algorithm PFSM [34]. Although the open-accessed simulations from the WaveWatch-III model have a lower spatial resolution of 0.5° grid, the model results can be used for independent validation purposes. We applied the CSAR_WAVE model to a total of 96/70 GF-3 SAR images in VV/HH polarization and then compared the retrieval results with those from the WaveWatch-III model at 0.5 m bins of SWH. Figure 8 shows the SI of SWH is 36% with a 0.74 m RMSE for VV-polarization and the SI of SWH is 37% with a 0.74 m RMSE for HH-polarization.

As a result, it is found that SAR-derived winds have a good agreement with ECMWF re-analysis winds for wind speeds between 5 and 10 m/s. However, SAR-derived winds are larger than ECMWF re-analysis winds for wind speeds smaller than 5 m/s and wind speeds greater than 10 m/s. We think it is probably due to the change of atmospheric stability at low winds and high wind conditions. Compared with SWH from WaveWatch-III model, the SWH retrieval results are generally larger. It is necessary to figure out that herein statistical results have a larger error than the result as exhibited in Figure 6, due to the coarse spatial resolution of wave data from WaveWatch-III model. To give a better performance, the bias can somehow be improved with the simple expediency of subtracting 0.6 for VV-polarization or 0.7 for HH-polarization.

## 6. Conclusions

Wind speed and SWH are two of most the important parameters in oceanography research, and SAR has the capability to observe wind and wave in wide spatial coverage. The Chinese GF-3 satellite carries a C-band SAR sensor, and has been operating in 12 imaging modes with a fine spatial resolution of up to 1 m since September 2016. Recently, the validation of SAR-derived winds from GF-3 images has been presented in [50]. In our work, the accuracy of wind speed and SWH retrieval results from GF-3 SAR have been simultaneously investigated.

We employed wind directions directly from ECMWF re-analysis data. Then CMOD5N, together with the PR model, involving the dependence on wind speed and incidence angle, were used for retrieving wind speeds. Validations against NDBC in situ buoys showed a 1.4 and 1.9 m/s RMSE of wind speed through 16 VV-polarization and 42 HH-polarization GF-3 SAR images. SAR-derived wind speeds from an additional 96/70 GF-3 images in VV/HH polarization were compared with ECMWF re-analysis winds, showing a 2.0/2.2 m/s RMSE of wind speed, respectively.

Theoretical-based algorithms and empirical CWAVE models have been developed in recent years. However, all of these rely on either wind speed or computations from numeric wave models. The new empirical CSAR_WAVE model is designed based on the relation between SAR cutoff wavelength in azimuth direction and SWH, and can be applicable without any prior information. The comparisons between SWH retrieval results and measurements from NDBC in situ buoys show a 24 and 23% SI of SWH with a 0.58 and 0.57 m RMSE of SWH for VV-polarization and HH polarization, respectively. We also compared the SWH retrieval results with the computations from the WaveWatch-III model, showing a 36/37% SI of SWH through 96/70 GF-3 images in VV/HH polarization. Therefore, CSAR_WAVE is suitable for wave retrieval from Chinese C-band GF-3 SAR.

In summary, the proposed methods are operationally applicable for wind and wave retrieval from C-band GF-3 SAR images in co-polarization. Moreover, the independent extraction of wind and wave from co-polarization GF-3 SAR images due to SAR-derived wind speed is no longer required in the process of wave retrieval when using the empirical CSAR_WAVE model. It is concluded that the performance achieves the design requirements of GF-3 SAR referred to the preliminary assessment of winds and waves presented in this study.
